# Psoas Muscle Volume Is a Useful Predictor of Postoperative Outcome in Elderly Patients With Non‐Small Cell Lung Cancer

**DOI:** 10.1111/1759-7714.70077

**Published:** 2025-04-28

**Authors:** Shinogu Takashima, Tsubasa Matsuo, Shoji Kuriyama, Hidenobu Iwai, Haruka Suzuki, Tatsuki Fujibayashi, Sumire Shibano, Yusuke Sato, Kyoko Nomura, Yoshihiro Minamiya, Kazuhiro Imai

**Affiliations:** ^1^ Department of Thoracic Surgery Akita University Graduate School of Medicine Akita Japan; ^2^ Department of Health Environmental Science and Public Health Akita University Graduate School of Medicine Akita Japan

**Keywords:** aged, lung neoplasms, postoperative complications, sarcopenia

## Abstract

**Background:**

As the population ages, the number of elderly lung cancer patients has been increasing. While surgery is the best treatment for resectable lung cancer, elderly patients often have multiple comorbidities, making accurate preoperative risk assessment crucial when formulating an appropriate treatment plan. This study aims to explore how psoas muscle volume relates to postoperative outcomes in elderly lung cancer patients.

**Methods:**

This single‐center, retrospective study included 344 elderly (≥ 75) patients who underwent complete surgical resection for non‐small cell cancer between 2010 and 2023. The psoas muscle volume index (PVI, cm^3^/m^3^) was measured using a 3‐dimensional imaging workstation based on preoperative computed tomography images and grouped based on the median value for each gender. Postoperative complications and survival rates were then compared between the groups.

**Results:**

The median PVI was 60.5 cm^3^/m^3^ for males and 47.7 cm^3^/m^3^ for females. The PVI‐high group had significantly fewer complications (15.6%) than the PVI‐low group (37.1%) (*p* < 0.001). The 5‐year overall survival (OS) rate was higher in the PVI‐high group (80.5%) than in the PVI‐low group (66.7%) (*p* = 0.01). Multivariate analyses showed that PVI‐high was an independent predictor of lower complication risk (odds ratio 0.28, *p* < 0.001) and an independent factor that improved OS (hazard ratio 0.60, *p* = 0.042).

**Conclusions:**

PVI in elderly lung cancer patients is associated with postoperative complications and survival.

## Graphical abstract

Preoperative psoas muscle volume in elderly lung cancer patients is associated with postoperative complications and survival. Screening for sarcopenia using psoas muscle volume may help to avoid unfavorable outcomes in these patients.

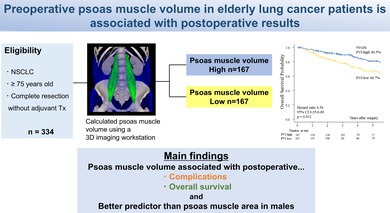

**Abbreviations**
3D: three‐dimension
AUC: area under curve
CCI: Charlson comorbidity index
CD: Clavien‐Dindo classification
CGA: Comprehensive Geriatric Assessment
CI: confidence interval
CT: computed tomography
NSCLC: non‐small cell lung cancer
OS: overall survival
PA: psoas muscle area
PAI: psoas muscle area index
PET: positron emission tomography
PPV: positive predictive value
PS: performance status
PV: psoas muscle volume
PVI: psoas muscle volume index
ROC: receiver operating characteristic

## 1 Background

Lung cancer is a leading cause of death worldwide [1], and as populations age, opportunities to treat elderly lung cancer patients increase. For patients of all ages, surgery is the best option for treating resectable cancers, but because elderly patients often have a variety of comorbidities, an accurate preoperative risk assessment is essential when formulating a treatment plan.

Sarcopenia, which was also directly associated with undernutrition or undernutrition risk, is defined as age‐related loss of muscle mass accompanied by low muscle strength and low physical performance [2, 3]. Notably, sarcopenia is reportedly associated with various cancer prognoses. A recent meta‐analysis showed that sarcopenia is associated with longer hospital stays and a higher incidence of total postoperative mortality in colorectal cancer patients [4], increased postoperative complications in gastric cancer patients [5], and poorer postoperative survival in nonsmall cell lung cancer (NSCLC) patients [6]. Besides, in resected NSCLC, sarcopenia was associated with increasing age, being male, smoking habit, lower body mass index, and postoperative major complications [7, 8]. If the sarcopenia is known using imaging modalities before surgery, the appropriate postoperative management can be provided. According to systematic reviews and meta‐analyses in NSCLC [6, 9], geriatric nutritional risk can lead to poorer survival. Although several nutritional biomarkers such as the Prognostic Nutritional Index and Controlling Nutritional Status were applied to predict the survival of NSCLC patients, these indices lacked worth of use in elderly patients because of limitations in usual weight estimation [9].

The methods used to diagnose sarcopenia and cutoff values for skeletal muscle mass vary from study to study, and a diagnosis of sarcopenia is generally based on skeletal muscle mass determined using dual‐energy X‐ray absorptiometry or bioimpedance methods [10]. However, most studies in the oncology field use computed tomography (CT) imaging to identify sarcopenia. In cancer patients, moreover, it is also helpful to evaluate CT images acquired in clinical practice. Measuring the psoas muscle area (PA) at the level of the third lumbar vertebra is widely used to evaluate muscle mass with CT [7, 8]. On the other hand, a novel method in which bilateral psoas muscle volume (PV) is measured using a three‐dimensional (3D) imaging workstation has been reported [11, 12, 13]. A PV index (PVI) reflects the general condition of cancer patients, especially their nutritional status. To our knowledge, there have been no studies investigating the relationship between PV and postoperative outcomes in elderly lung cancer patients.

In the present study, therefore, we aimed to clarify the relationship between PV and postoperative outcomes in elderly (≥ 75 years) lung cancer patients.

## 2 Materials and Methods

### 2.1 Ethical Requirements

The experimental protocols used in this study were approved by the institutional review board at Akita University Hospital under approval number 2679. All data were collected in accordance with this IRB Protocol.

### 2.2 Patients

This was a single‐center, retrospective study of patients ≥ 75 years of age who had undergone complete surgical resection for NSCLC without neoadjuvant therapy at Akita University Hospital between January 2010 and March 2023. Of the 346 patients treated during that period, 344 were included in this study. Two patients for whom CT or positron emission tomography‐CT (PET/CT) of the pelvic region taken within 3 months before surgery could not be obtained were excluded. The patients' characteristics are listed in Table 1.

**TABLE 1:** Patients characteristics.

	*n* = 334
Age (years)	78 (75–87)
ECOG performance status	
0/1/2	265 (79)/66 (20)/3 (1)
Smoking status	
Never/ever or current	142 (42)/192 (58)
Charlson Comorbidity Index score	
0–1/2 or more	195 (68)/139 (32)
Glasgow prognostic score	
0/1/2	310 (93)/20 (6)/4 (1)
Prognostic Nutritional Index	49.4 (36.1–70.5)
Neutrophil to lymphocyte ratio	2.35 (0.54–49.12)
Body Mass Index	22.4 (15.5–32.7)
Surgical procedure	
Segmentectomy	77 (23)
Lobectomy	250 (75)
Biloectomy	5 (1)
Pneumonectomy	2 (1)
Tumor size (mm)	24 (3–135)
Nodal status	
pN0 / N1 / N2	298 (89)/17 (5)/19 (6)
Pathological stage	
0/I/II/III	16 (5)/237 (71)/52 (16)/29 (8)
Histology	
Adenocarcinoma	253 (76)
Squamous cell carcinoma	74 (22)
Others	7 (2)
Adjuvant therapy	53 (16)
EGFR/ALK mutation positive	49 (15)

*Note:* Values are presented as median (range) or number (%).

Abbreviations: ALK, anaplastic lymphoma kinase; EGFR; epidermal growth factor receptor.

### 2.3 Image Analysis of Psoas Muscle Volume

**FIGURE 1:**
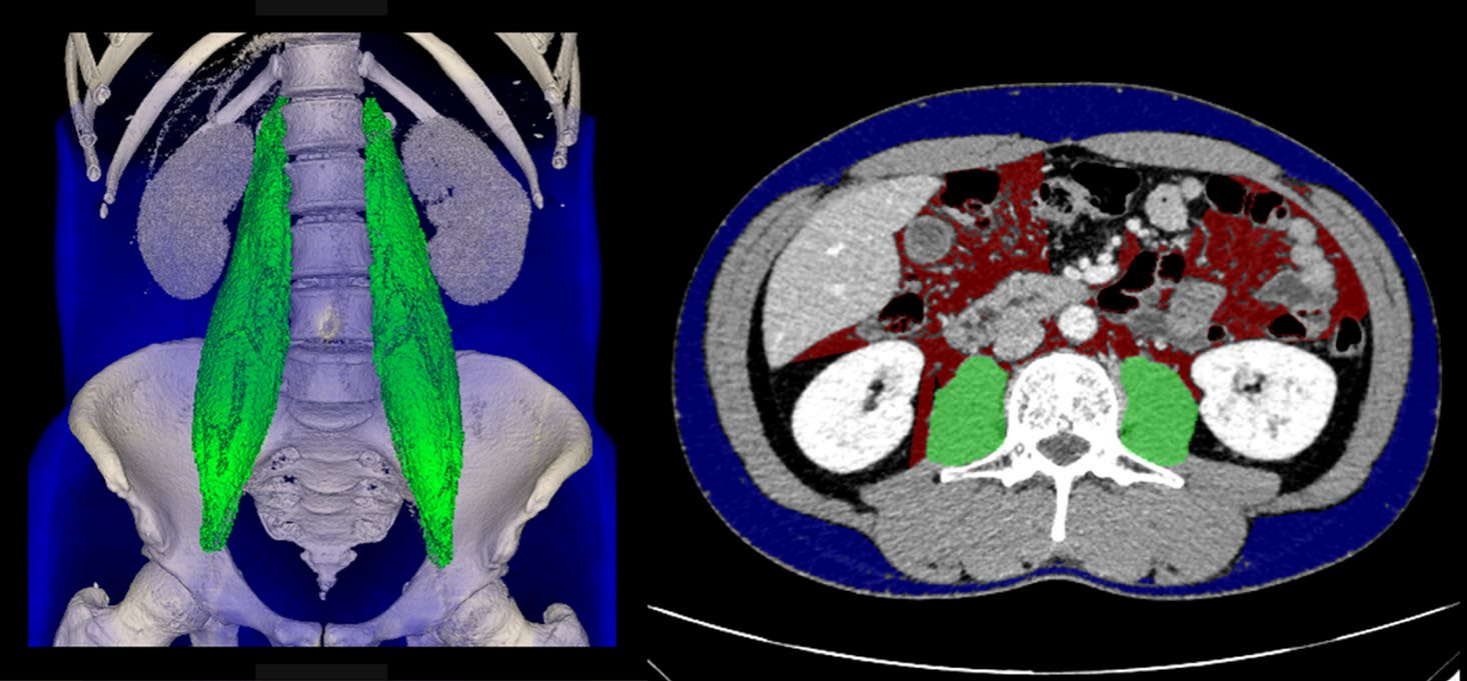
Bilateral psoas major muscle volumes measured semiautomatically using image analysis software (FUJIFILM SYNAPSE VINCENT; Fujifilm Medical, Tokyo, Japan).

**FIGURE 2:**
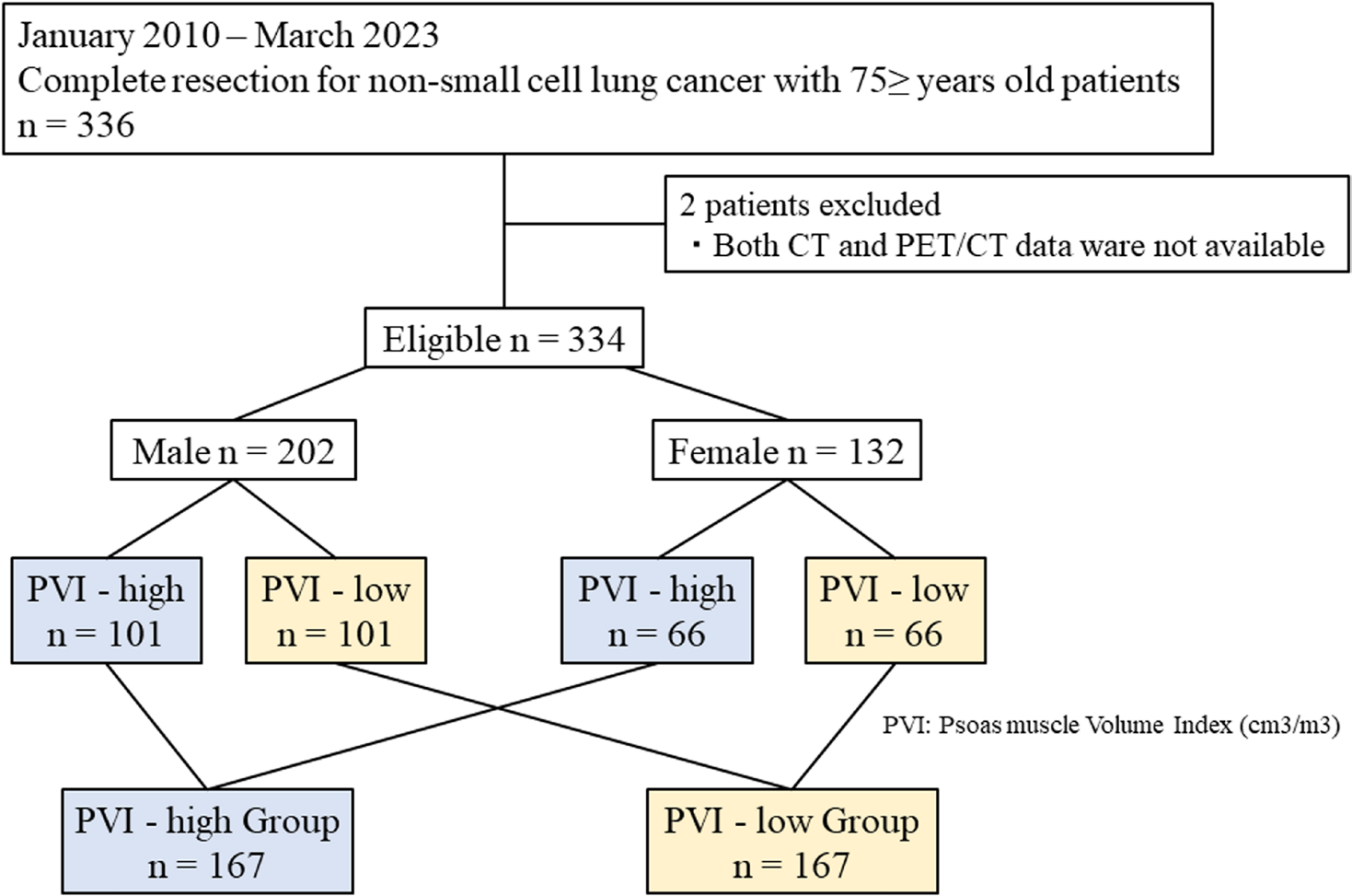
Flow chart illustrating the subject enrollment protocol. Patients were grouped by gender based on the median PVI value.

Bilateral PV (cm^3^) was calculated using a 3D imaging workstation (SYNAPSE VINCENT; Fujifilm Corp, Tokyo, Japan) based on preoperative CT or PET/CT (Figure 1). As described previously [11, 13], we standardized PV by dividing it by the cube of the height (m^3^) to generate a PVI for analysis. Because a standard cutoff value for PVI has not been established and based on a report that about half of lung cancer patients have sarcopenia [14], we divided the patients by gender into PVI‐high and PVI‐low groups based on the median PVI value. A diagram of the process used for case selection and allocation is shown in Figure 2.

### 2.4 Surgical Procedure and Follow‐Up

All patients received radical lung resection plus systemic lymph node dissection after a standard preoperative examination. Pathological staging was based on the 8th edition of the UICC‐TNM classification [15]. Although the follow‐up schedule after surgery varied, it usually entailed a chest CT every 6 or 12 months for the first five years. If recurrence was suspected, the follow‐up schedule was tightened. Postoperative complications within 90 days after surgery were assessed using the Clavien‐Dindo classification (CD) [16].

### 2.5 Statistical Analysis

The clinical characteristics of the PVI‐high and PVI‐low groups were compared using the Student *t*‐test or the Mann–Whitney *U* test for continuous variables and the Chi‐squared test or Fisher's exact test for categorical variables.

Univariate and multivariate logistic regression analyses were used to assess the relationship between PVI and postoperative complications (CD ≥ 2), and odds ratios with 95% confidence intervals (CIs) were calculated. The areas under receiver operating characteristic (ROC) curves (AUCs) were compared using DeLong's test. The sensitivity, specificity, positive predictive value, negative predictive value, accuracy, and AUC were all calculated using standard formulas with a 2 × 2 table of the collected data. 95% CIs were calculated using the Clopper‐Pearson method.

Kaplan–Meier curves were used to compare overall survival (OS) in the PVI‐high and PVI‐low groups. Differences between the survival curves were assessed using the log‐rank test. Multivariate analysis of OS was performed using the Cox proportional hazards regression model, and hazard ratios along with 95% CIs were calculated. All statistical analyses and drawings of the ROC curves were performed using JMP IN 17.0.0 software (SAS Institute, Cary, NC, USA). Values of p were 2‐sided and considered significant if less than 0.05.

## 3 Results

**FIGURE 3:**
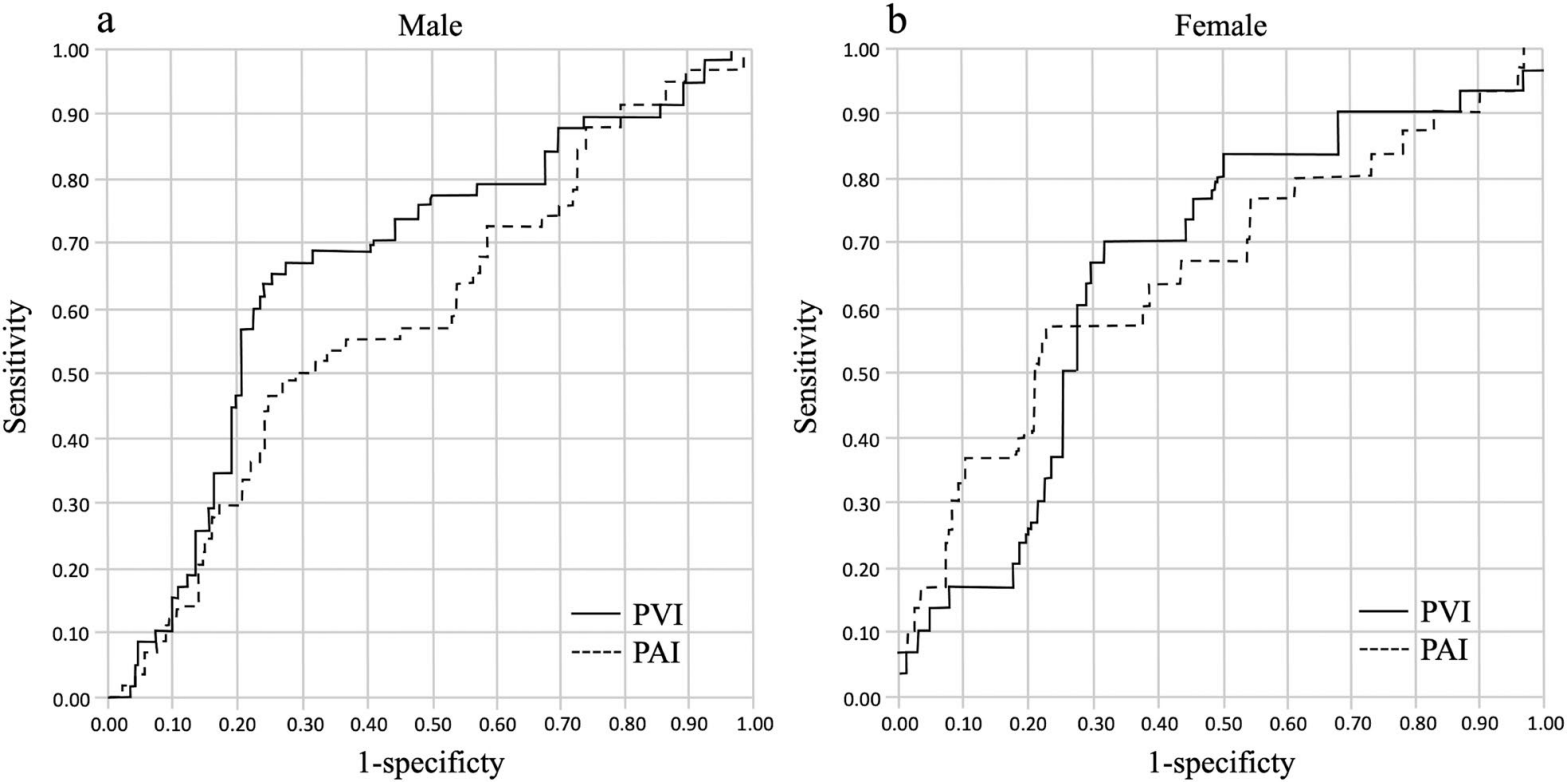
Receiver operating characteristic (ROC) curves for predicting postoperative complications (CD≧ 2) using PVI or PAI. Panel (a) shows males and panel (b) shows females; solid and dashed lines indicate PVI and PAI, respectively.

We evaluated whether PV or PA was a better predictor of postoperative complications. As with PV, PA was calculated semi‐automatically using an imaging workstation and normalized by dividing the PA value at the level of the third lumbar vertebra by the square of height (m^2^) to obtain a PA index (PAI). ROC curves were then used to determine the cutoff values for PVI and PAI that yielded the highest combined sensitivity and specificity for predicting postoperative complications in males and females. The ROC curves are shown in Figure 3, and sensitivity, specificity, positive predictive values, negative predictive values, accuracy, and AUC for each are shown in Table 2. In males, in particular, the PVI was a better predictor of the incidence of postoperative complications than the PAI (*p* = 0.021, DeLong's test).

**TABLE 2:** Prediction of postoperative complications (CD ≥ 2) using PVI and PAI for each gender.

	Male		*p*	Female		*p*
Method	PVI	PAI		PVI	PAI	
Ideal cutoff value (PVI: cm3/m3, PAI: cm2/m2)	55.6	4.2		44.2	3.1	
Sensitivity, % (95% CI)	65.5 (51.9–77.5)	46.6 (33.3–60.1)		70.0 (50.6–85.3)	56.7 (37.4–74.5)	
Specificity, % (95% CI)	74.3 (66.4–81.2)	75.0 (67.1–81.8)		68.6 (58.7–77.5)	76.5 (67.0–84.3)	
PPV, % (95% CI)	50.7 (38.9–62.4)	42.9 (30.5–56.0)		39.6 (26.5–54.0)	41.5 (26.3–57.9)	
NPV, % (95% CI)	84.3 (76.7–90.1)	77.7 (69.9–84.3)		88.6 (79.5–94.7)	85.7 (76.8–92.2)	
Accuracy, % (95% CI)	71.8 (65.0–77.9)	66.8 (59.9–73.3)		68.9 (60.3–76.7)	72.0 (63.5–79.4)	
AUC (95% CI)	0.669 (0.585–0.754)	0.585 (0.499–0.673)	0.021	0.660 (0.549–0.771)	0.648 (0.525–0.770)	0.770

Abbreviations: AUC, area under curve; NPV, negative predictive value; PAI, psoas muscle area index; PPV, positive predictive value; PVI, psoas muscle volume index.

The median PVIs for males and females were 60.5 cm^3^/m^3^ and 47.7 cm^3^/m^3^ respectively. The clinical characteristics of PVI‐high and PVI‐low groups are compared in Table S1. The median follow‐up duration was 3.46 years (range 0.03–11.36 years) for all patients.

The incidence of postoperative complications (CD ≥ 2) among all the patients was 26.3%. A detailed breakdown of the postoperative complications is shown in Table S2. The PVI‐high group had significantly fewer postoperative complications than the PVI‐low group (15.6% vs. 37.1%; *p* < 0.001, Chi‐squared test). For univariate and multivariate logistic regression analyses performed to assess risk factors for postoperative complications, gender, age, Brinkman index, PS, CCI, ppo‐FEV_1.0_, clinical stage, surgical approach, extent of resection, duration of surgery, and PVI were included as factors in each analysis. In the univariate analysis, poor PS (odds ratio, 2.53; 95% CI, 1.45–4.43; *p* = 0.001) and high CCI (odds ratio, 1.28; 95% CI, 1.07–1.52; *p* = 0.006) were associated with an increased risk of complications, while PVI‐high (odds ratio, 0.31; 95% CI, 0.19–0.53; *p* < 0.001) was associated with a decreased risk. Multivariate analysis showed PVI‐high to be an independent factor affecting the likelihood of postoperative complications (odds ratio, 0.28; 95% CI, 0.16–0.50; *p* < 0.001) (Table 3).

**TABLE 3:** Univariate and multivariate logistic regression analysis for postoperative complications (CD ≥ 2).

	Postoperative complication		Univariate analysis				Multivariate analysis			
	(+)	(−)	Odds ratio	95% CI		*p*	Odds ratio	95% CI		*p*
Gender (male)	58 (65.9)	144 (58.5)	1.37	0.82	2.28	0.221	0.73	0.32	1.68	0.463
Age (years)	78.6 ± 2.8	78.7 ± 3.0	0.99	0.34	2.50	0.874	0.94	0.86	1.04	0.234
Brinkman index	735.1 ± 857.9	547.8 ± 719.9	1.00	0.99	1.01	0.088	1.00	0.99	1.00	0.413
Performance status (≥ 1)	29 (33.0)	40 (16.3)	2.53	1.45	4.43	0.001	2.43	1.30	4.57	0.005
Charlson comorbidity index	1.7 ± 1.4	1.2 ± 1.3	1.28	1.07	1.52	0.006	1.28	1.04	1.58	0.021
ppo‐FEV1.0	1742.9 ± 459.0	1685.3 ± 415.5	1.00	0.99	1.01	0.282	1.00	0.99	1.01	0.524
Clinical stage (≥IB)	37 (42.1)	87 (35.4)	1.33	0.81	2.18	0.268	1.76	0.96	3.22	0.069
Open‐thoracotomy/VATS	23 (26.1)	61 (24.8)	1.07	0.62	1.87	0.804	0.81	0.42	1.55	0.529
Segmentectomy/Lobectomy	24 (27.3)	53 (21.5)	1.37	0.78	2.39	0.280	1.65	0.84	3.24	0.147
Duration of surgery > 180 min	65 (73.9)	164 (66.7)	1.41	0.82	2.44	0.207	1.70	0.91	3.16	0.093
PVI (high)	26 (29.6)	141 (57.3)	0.31	0.19	0.53	< 0.001	0.28	0.16	0.50	< 0.001

*Note:* Values are presented as mean ± standard deviation or number (%).

Abbreviations: ppo‐FEV1.0, predictive postoperative forced expiratory volume in one second; PVI, psoas muscle volume index; VATS, video‐assisted thoracic surgery.

**FIGURE 4:**
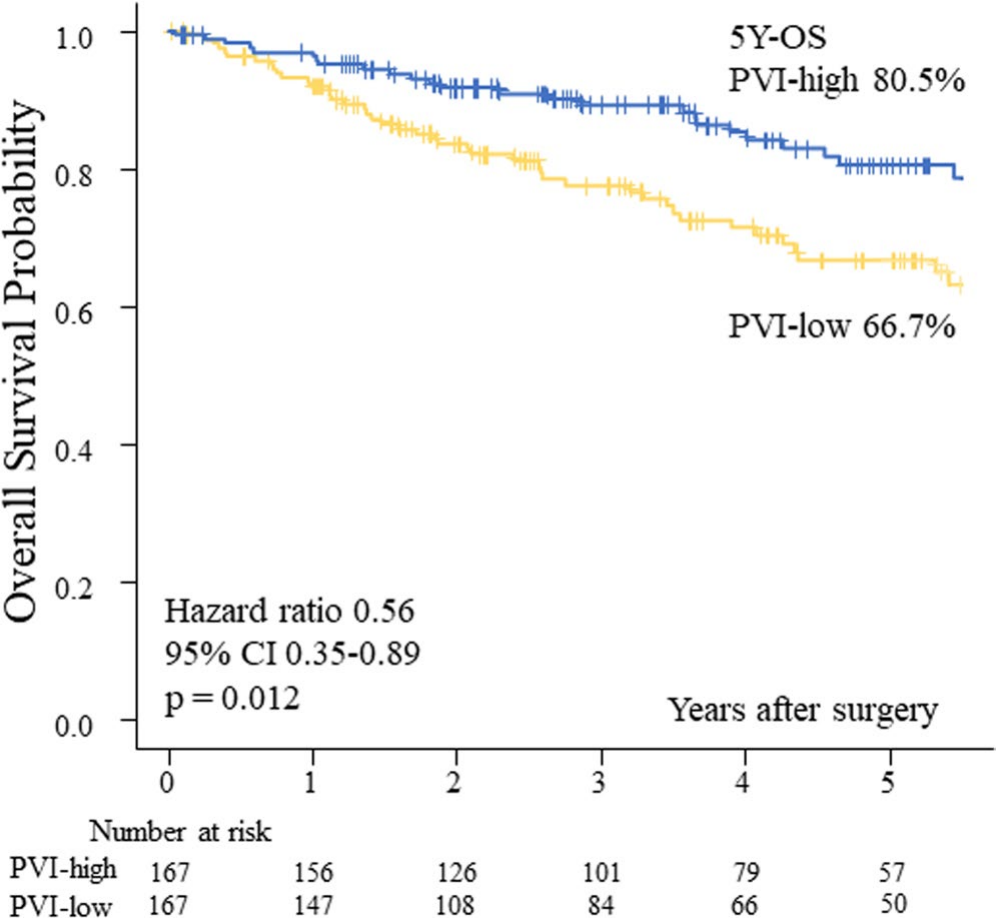
Kaplan–Meier curves comparing 5‐year overall survival between patients with PVI‐high and PVI‐low.

The Kaplan–Meier curves for OS in the PVI‐high and PVI‐low groups are shown in Figure 4. The 5‐year OS rate in the PVI‐high group was significantly higher than in the PVI‐low group (80.5% vs. 66.7%; *p* = 0.012, log‐rank test). In a multivariate Cox proportional hazard analysis to determine the factors affecting OS, the factors considered were gender, age, CCI, postoperative complication, pathological stage, histological type, adjuvant therapy, and PVI. Among those, pathological stage (hazard ratio, 1.73; 95% CI, 1.47–2.03; *p* < 0.001) and PVI‐high (hazard ratio, 0.60; 95% CI, 0.36–0.98; *p* = 0.042) were independent factors affecting OS (Table 4).

**TABLE 4:** Univariate and multivariate cox proportional hazard analysis for overall survival after surgery.

	Univariate analysis				Multivariate analysis			
	Hazard ratio	95% CI		*p*	Hazard ratio	95% CI		*p*
Gender (male)	2.27	1.35	3.81	0.002	1.65	0.93	2.92	0.088
Age	1.03	0.95	1.11	0.485	0.96	0.88	1.05	0.359
Charlson Comorbidity Index	1.19	1.03	1.36	0.022	1.12	0.95	1.31	0.190
Postoperative complication (CD ≥ 2)	1.67	1.02	2.75	0.041	1.14	0.67	1.95	0.624
Pathological stage	1.66	1.43	1.93	< 0.001	1.73	1.47	2.03	< 0.001
Histological type (adeno)	0.48	0.30	0.78	0.003	0.79	0.47	1.33	0.376
Adjuvant therapy (presence)	1.17	0.68	2.00	0.573	0.56	0.31	1.03	0.062
PVI (high)	0.56	0.35	0.89	0.012	0.60	0.36	0.98	0.042

Abbreviation: PVI, psoas muscle volume index.

## 4 Discussion

In the present study, preoperative PVI was found to be associated with postoperative complications and survival in elderly (≥ 75 years) lung cancer patients. PVI was also a better predictor of postoperative complications than PAI in males.

With increases in life expectancy and aging of the population, we expect to be treating larger numbers of elderly lung cancer patients in the future. According to the annual report of the Japanese Association for Thoracic Surgery, of the 46 624 lung cancer surgeries performed in Japan in 2021, 65% were performed on patients over 70 years old and 15% on patients over 80 years old, and this percentage is increasing every year [17]. Several reports have shown that age itself is not a significant risk factor for surgery [18, 19, 20, 21, 22, 23, 24], and the third edition of the American College of Chest Physicians evidence‐based guidelines recommends that surgery should not be refused based on age alone [25]. Thus, a key challenge for thoracic surgeons is how to determine the optimal treatment for this heterogeneous group of patients, taking into consideration comorbidities, physical reserve, cognitive function, and disability. One solution to this challenge would be a comprehensive geriatric assessment (CGA). The Italian Society of Geriatrics and Gerontology recommends that cognitive status, functional status, sarcopenia, nutritional status, psychological state, comorbidities, quality of life, and social status be evaluated in a CGA for elderly cancer patients [26]. Although performing a CGA is time‐consuming, tools have been developed for simple assessment, and our institution has introduced a CGA for prethoracic surgery patients over 65 years of age.

Sarcopenia is defined as age‐related progressive loss of skeletal muscle mass and function. The diagnostic criteria for sarcopenia are still evolving. Initially, the focus was solely on the loss of skeletal muscle mass. However, it has become clear that decreased muscle function is more associated with outcomes such as falls, hospitalization, and death [27, 28], and diagnostic criteria combining skeletal muscle mass and muscle function have been developed by both the European Working Group on Sarcopenia [29] and the Asian Working Group for Sarcopenia [10]. Specifically, in the AWGS2019, low muscle strength was defined as handgrip strength < 28 kg for men and < 18 kg for women, low physical performance defined as a 6‐min walk at < 1.0 m/s, a Short Physical Performance Battery score ≤ 9, or a 5 chair stand test ≥ 12 s, as well as a decline in muscle mass of < 7.0 kg/m^2^ in men and < 5.4 kg/m^2^ in women, determined using dual‐energy X‐ray absorptiometry, or by a decline of < 7.0 kg/m^2^ in men and < 5.7 kg/m^2^ in women, determined using bioimpedance [10]. Cancer patients have been shown to undergo various changes in body composition, with alterations in the relative amounts of muscle and fat and in bone density [30]. The presence of sarcopenia reportedly worsens prognosis in a variety of cancers [4, 5, 31, 32] and is associated with increased depression, decreased quality of life [33], and increased risk of cardiovascular disease in cancer patients [34]. However, the mechanism by which sarcopenia worsens prognosis, especially in early‐stage cancer patients, remains unclear.

CT scans are routinely used in clinical practice, and their use for estimating skeletal muscle mass in patients with lung cancer is reasonable. Used most commonly is the cross‐sectional area of the skeletal muscle mass at the level of the third lumbar vertebra [8, 35, 36, 37, 38, 39, 40, 41]. This is because the distribution of fat and skeletal muscle mass there strongly correlates with total body fat and skeletal muscle mass [42]. On the other hand, because skeletal muscle mass has sometimes been measured and evaluated at the level of the fourth thoracic vertebra [43, 44], further research was required to determine the optimal level of CT scanning for evaluating sarcopenia. The semi‐automatic and highly reproducible method in which PVI is measured using a 3D imaging workstation could answer that question, and we suggested PVI is a better indicator than muscle area. To our knowledge, this is the first report to focus on PV in elderly lung cancer patients and to show a significant relationship between PV and both postoperative complications and OS.

There are several limitations to this study. First, it is a single‐center, retrospective study with a small sample size, and the potential for selection bias could not be eliminated. Second, the PVI cutoff value for determining the PVI‐high and PVI‐low groups was the median, taking gender into consideration; a standard threshold value for PVI has yet to be established. Third, PVI semi‐automatically calculated using a 3D imaging workstation was reproducible, but psoas muscle functionality was not assessed. Up to now, the field of cancer research has relied solely on measuring skeletal muscle mass when assessing sarcopenia, and there is a need for integration of muscle strength into future analyses. Fourth, wedge resection was occasionally selected instead of segmentectomy/lobectomy in daily clinical practice for elderly patients with comorbidities. In early‐stage NSCLC patients aged ≥ 80 years, OS and recurrence‐free survival curves were reported similar among three procedures (lobectomy/segmentectomy/wedge resection) [45]. However, patients who underwent wedge resection were not included in this study, and their PVI remains unclear.

In conclusion, our results show that preoperative psoas muscle volume in elderly lung cancer patients is associated with postoperative complications and survival. Screening for sarcopenia using psoas muscle volume in addition to other preoperative assessments may help to avoid unfavorable outcomes in these patients.

## Author Contributions

All authors had full access to the data in the study and took responsibility for the integrity of the data and the accuracy of the data analysis. Conceptualization: S.T. and K.I. Methodology: S.T., K.I. Investigation: S.T., T.M., S.K., H.I., H.S., T.H., and S.S. Formal analysis: S.T. and K.N. Writing – original draft: S.T. and K.I. Writing – review and editing: T.M., S.K., H.I., H.S., and Y.M. Supervision: Y.M.

## Ethics Statement

The experimental protocols for this study were approved by the institutional review board at Akita University Hospital under approval number 2679. All data were collected in accordance with this IRB Protocol.

## Consent

Comprehensive written informed consent was obtained from all patients.

## Conflicts of Interest

The authors declare no conflicts of interest.

## Supporting information


**Table S1.** Patients characteristics.
**Table S2.** Distribution of postoperative complications (CD ≥ 2).

## Data Availability

The datasets used and/or analyzed during the current study are available from the corresponding author upon reasonable request.
