# Supplementary material for: Psoas Muscle Volume Is a Useful Predictor of Postoperative Outcome in Elderly Patients With Non‐Small Cell Lung Cancer
Source: Thorac Cancer. 2025 Apr 28;16(8):e70077. doi: 10.1111/1759-7714.70077 (PMC12035415; doi:10.1111/1759-7714.70077)
Supplement: Supplementary file 1 — Table S1. Patients characteristics. Table S2. Distribution of postoperative complications (CD ≥ 2). [file TCA-16-e70077-s001.docx]

**Supplementary Table 1. Patients characteristics**

|  | PVI-high (n = 167) | PVI-low (n = 167) | *P* |
| --- | --- | --- | --- |
| Age (years) | 78 （75-87） | 79 （75-87） | 0.006 |
| ECOG performance status |  |  | 0.145 |
| 0 / 1 / 2 | 137 (82) / 30 (20) / 0 | 128 (77) / 36 (21) / 3 (2) |  |
| Smoking status |  |  | 1 |
| Never / Ever or Current | 96 (57) / 71 (43) | 96 (57) / 71 (43) |  |
| Charlson Comorbidity Index score |  |  | 0.824 |
| 0-1 / 2 or more | 99 (59) / 68 (41) | 96 (57)/ 71 (43) |  |
| Glasgow Prognostic Score |  |  | 0.383 |
| 0 / 1 / 2 | 158 (94) / 7 (5) / 2 (1) | 152 (91) / 13 (8) / 2 (1) |  |
| Prognostic Nutritional Index | 50.0 (37.5-65.2) | 48.6 (36.1-70.5) | 0.003 |
| Neutrophil to Lymphocyte Ratio | 2.24 (0.54-49.12) | 2.45 (0.82-14.71) | 0.024 |
| Body Mass Index | 24 (15.5-32.7) | 21.4 (15.7-32.5) | <0.001 |
| Surgical procedure |  |  | 0.972 |
| Segmentectomy | 38 (23) | 39 (23) |  |
| Lobectomy | 126 (75) | 124 (74) |  |
| Biloectomy | 2 (1) | 3 (2) |  |
| Pneumonectomy | 1 (1) | 1 (1) |  |
| Tumor size (mm) | 25 (5-120) | 24 (3-135) | 0.473 |
| Nodal status |  |  |  |
| pN0 / N1 / N2 | 146 (87) / 9 (5)/12 (7) | 152 (91) / 8 (5) / 7 (4) |  |
| Pathological stage |  |  | 0.285 |
| 0 / I / II / III | 8 (5) / 123 (74) / 22 (13) / 14 (8) | 8 (5) / 114 (77) / 30 (18) / 15 (9) |  |
| Histology |  |  | 0.686 |
| Adenocarcinoma | 125 (75) | 128 (77) |  |
| Squamous cell carcinoma | 38 (23) | 36 (21) |  |
| Other | 4 (2) | 3 (2) |  |
| Adjuvant therapy | 33 (20) | 20 (12) | 0.071 |
| EGFR / ALK mutation positive | 31 (18) | 18 (11) | 0.062 |

Values are presented as median (range) or number (%).

PVI: Psoas muscle Volume Index, EGFR: Epidermal Growth Factor Receptor, ALK: Anaplastic Lymphoma Kinase

**Supplementary Table2. Distribution of postoperative complications (CD≥2)**

|  | PVI-high (n = 167) |  | PVI-low (n = 167) | *P* |
| --- | --- | --- | --- | --- |
| Overall | 26 (15.6) |  | 62　(37.1) | ＜0.001 |
| Prolonged air leak | 6 (3.6) |  | 14 (8.4) |  |
| Bacterial pneumonia | 7 (4.2) |  | 12 (7.2) |  |
| Acute exacerbation of interstitial pneumonia | 0 |  | 4 (2.4) |  |
| Empyema | 5 (3.0) |  | 3 (1.8) |  |
| Bronchopleural fistula | 0 |  | 1 (0.6) |  |
| Respiratory failure | 1 (0.6) |  | 2 (1.2) |  |
| Postoperative bleeding | 1 (0.6) |  | 4 (2.4) |  |
| Chylothorax | 2 (1.2) |  | 6 (3.6) |  |
| Postoperative atrial fibrillation | 5 (3.0) |  | 9 (5.4) |  |
| Ischemic heart disease | 0 |  | 4 (2.4) |  |
| Cerebral infarction | 0 |  | 3 (1.8) |  |
| Renal infarction | 1 (0.6) |  | 0 |  |
| Pulmonary infarction | 0 |  | 1 (0.6) |  |
| Others | 3 (1.8) |  | 11 (6.6) |  |

Values are presented as number (%)

PVI: Psoas muscle Volume Index
